# Rose Bengal–Chitosan Nanocomposites for Oral Administration

**DOI:** 10.3390/nano15100706

**Published:** 2025-05-08

**Authors:** Sara Demartis, Camila J. Picco, Octavio E. Fandiño, Eneko Larrañeta, Ryan F. Donnelly, Paolo Giunchedi, Giovanna Rassu, Elisabetta Gavini

**Affiliations:** 1 Department of Medicine, Surgery and Pharmacy, University of Sassari, 07100 Sassari, Italy; sdemartis@uniss.it (S.D.); grassu@uniss.it (G.R.); eligav@uniss.it (E.G.); 2 School of Pharmacy, Medical Biology Centre, Queen’s University Belfast, Belfast BT9 7BL, UK; cjpicco5@gmail.com (C.J.P.); o.fandino@qub.ac.uk (O.E.F.); e.larraneta@qub.ac.uk (E.L.); r.donnelly@qub.ac.uk (R.F.D.)

**Keywords:** Rose Bengal, chitosan, oral administration, nanocomposites, gastrointestinal release

## Abstract

Rose Bengal (RB) holds promise for therapeutic applications in the gastrointestinal (GI) tract but faces significant limitations due to poor bioavailability and stability in the GI environment. This in vitro proof-of-concept study aimed to develop an oral drug delivery system using self-assembled RB–chitosan (RBCS) nanocomposites formed via electrostatic interactions. RBCS nanocomposites exhibited high drug loading efficiency (87%) and a uniform particle size (~443 nm), with physicochemical analyses confirming molecular interactions and structural stability. However, in vitro studies revealed poor and highly variable drug release in simulated gastric fluids (SGFs), underlining the need for further optimization. To address these limitations, RBCS nanocomposites were encapsulated within well-established alginate beads (AlgBs). Among the tested systems, RBCS20-AlgBs were selected as the optimal one, forming a gastroresistant platform. Encapsulation mitigated burst release, enhanced structural integrity, and enabled sustained RB release under intestinal conditions. Swelling studies demonstrated that RBCS20-AlgBs maintained controlled hydration, preventing premature disintegration. Mathematical modeling indicated a matrix relaxation-driven release mechanism, with RBCS20-AlgBs demonstrating improved reproducibility compared to RB-loaded AlgBs (RB-AlgBs). Future studies should focus on evaluating in vivo performance to confirm the system’s efficacy for oral administration.

## 1. Introduction

Rose Bengal disodium salt (RB) is a synthetic xanthene dye with applications in diagnostics and therapeutics. Traditionally used as a staining agent in ophthalmology and a liver function test marker, RB may become highly relevant for its potential therapeutic application in the gastrointestinal (GI) tract, particularly in cancer therapy, antimicrobial treatments, and photodynamic therapy (PDT). In GI PDT, RB has demonstrated strong bactericidal effects against Enterobacteriaceae, including *E. coli*, suggesting its potential as an alternative treatment for infection and other GI bacterial diseases [1,2,3]. Additionally, RB has been incorporated into cellulose acetate films, achieving 100% eradication of *C. difficile*, underlining its role in GI pathogen control [4]. In GI cancers, particularly colorectal cancer, RB-based PDT has been optimized through nanotechnology-driven delivery systems, such as M13 bacteriophage conjugates, enhancing tumor penetration and photodynamic efficacy [5]. Beyond PDT, RB has demonstrated direct cytotoxic effects in gastric cancers, inducing oxidative stress and apoptosis in tumor cells even without light activation [6]. Additionally, RB has been explored as an immune system modulator in colorectal cancer [7] and as a chemosensitizer, improving the efficacy of chemotherapy drugs like doxorubicin when delivered via nanoparticle formulations [8]. These findings establish RB as a versatile therapeutic agent for GI diseases, though further research is needed to optimize its safety profile and clinical application.

Despite its potential, RB’s oral administration is significantly hindered by its physicochemical profile, resulting in poor bioavailability due to multiple absorption barriers in the GI tract. In the stomach, RB remains largely ionized in the acidic environment (pH 1–3), reducing its membrane permeability. As it transitions to the small intestine (pH 5–7), some RB molecules shift to a non-ionized form, improving absorption potential, but low lipophilicity limits its permeability across the intestinal epithelium [9,10,11]. Even when absorbed, RB undergoes extensive first-pass metabolism in the liver, leading to rapid biotransformation and biliary excretion, further reducing systemic drug availability. Additionally, toxicity studies indicate hepatic accumulation and metabolic alterations, raising concerns about long-term exposure, particularly in individuals with pre-existing liver conditions [12,13]. These challenges necessitate advanced formulation strategies to enhance RB’s absorption, stability, and therapeutic efficacy.

Biopolymers such as chitosan (CS) have shown great potential in overcoming RB’s poor bioavailability, enhancing its stability and permeability. A cationic polysaccharide derived from chitin, CS interacts ionically with negatively charged drugs, prolonging residence time at absorption sites while opening epithelial tight junctions, thereby enhancing systemic uptake. Additionally, CS-based drug formulations protect drugs from gastric acidity, enabling controlled intestinal release [14,15,16]. The combination of RB with CS has been studied for drug delivery, antimicrobial therapy, and cancer treatment, as CS enhances RB’s solubility, stability, and tissue retention, mitigating its rapid elimination and poor tumor accumulation [17]. RB-CS formulations are typically prepared as nanoparticle or chemical conjugates using ionic cross-linking agents such as sodium tripolyphosphate (TPP) or carbodiimide-based compounds [18,19,20,21]. However, some studies have explored milder preparation methods based on natural electrostatic interactions between RB’s anionic groups and the cationic amino groups of CS, enabling self-assembled nanoparticles under controlled pH conditions. This cross-linker free approach is considered more biocompatible and environmentally friendly [17]. Despite CS’s mucoadhesive and permeation-enhancing properties, existing RB-CS systems have focused almost exclusively on non-oral routes. Most published studies explored intravenous or localized administration for cancer PDT and sonodynamic therapy [17,20,21], or use RB-CS complexes for antimicrobial purposes, such as biofilm eradication and local infection control [18,19]. To date, no study has addressed the challenges of oral RB delivery using CS-based systems, representing a clear gap and an unmet need in the field of GI-targeted RB therapy.

**Figure 1:**
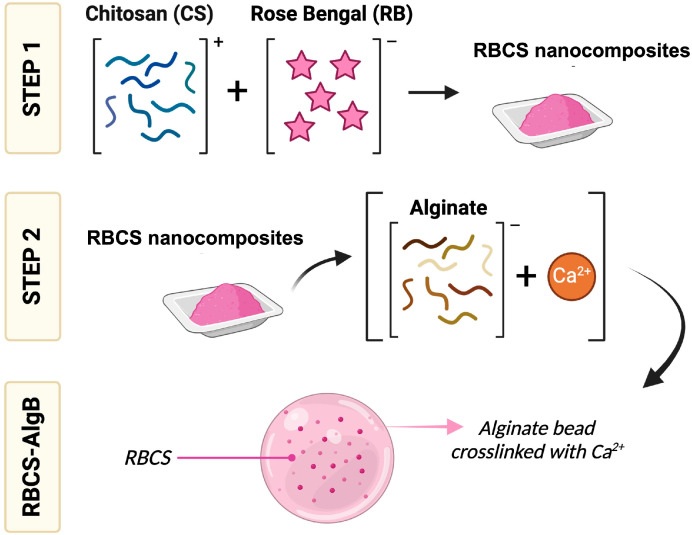
Schematic representation of the formulation process for RBCS nanocomposites encapsulated in AlgBs (RBCS-AlgBs). The process involves the self-assembly of RBCS nanocomposites via electrostatic interaction (Step 1), followed by encapsulation in AlgBs (Step 2) to enhance stability and enable controlled release for oral delivery. Created with Biorender.com.

This study aims to develop an oral drug delivery system for RB using RB-CS (RBCS) nanocomposites, addressing RB’s poor oral availability and enhancing its therapeutic applicability in the GI tract. The formulation strategy involved self-assembling RBCS nanocomposites through natural electrostatic interaction (Step 1), followed by encapsulation within established AlgBs (Step 2) to provide a gastroresistant system capable of ensuring targeted intestinal release (Figure 1). Unlike conventional CS-alginate systems, which typically rely on co-encapsulation of free drug and polymers, this approach integrates preformed RBCS nanocomposites into the alginate matrix, offering improved control over drug loading, matrix structure, and release kinetics. Particularly, the investigation focused on optimizing formulation parameters, characterizing physicochemical and technological properties, and evaluating in vitro drug release and swelling behavior of the developed systems.

## 2. Materials and Methods

### 2.1. Materials

Alginic acid sodium salt derived from *Macrocystis pyrifera* (kelp) with a viscosity of 2% in solution, glacial acetic acid (99.8%), calcium chloride, RB (dye content 95%), sodium taurocholate (STC), lecithin, pepsin, maleic acid, and sodium chloride were obtained from Sigma Aldrich (St. Louis, MO, USA). Sodium hydroxide was sourced from Carlo Erba Reagents (Cornaredo, Italy), and monobasic potassium phosphate from Merck (Darmstadt, Germany). Chitosan (CS) Chitoclear™ 1358 (MW = 103 kDa, deacetylation grade = 94%) was provided by PRIMEX EHF (Siglufjordur, Iceland). Tris (tris (hydroxymethyl) aminomethane), known as Trizma base, and anhydrous sodium acetate were also purchased from Sigma Aldrich (St. Louis, MO, USA).

### 2.2. Purification of CS

CS was purified using the method of Rassu et al. [22]. An amount of 2 g of CS was dissolved in 160 mL of 2% (*v*/*v*) aqueous acetic acid solution under magnetic stirring at room temperature for 4 h. The solution was boiled for 15 min to precipitate protein contaminants, centrifuged at 3000 rpm for 10 min (Eppendorf Centrifuge 5702R, Eppendorf, Milan, Italy), and the supernatant was decanted and filtered under vacuum using a 0.8 µm nitrocellulose membrane filter (Millipore, Darmstadt, Germany). The filtered solution was basified to pH 9 with 1M NaOH to precipitate the purified CS, which was then isolated by centrifugation (10 min, 3000 rpm) and washed three times with MilliQ water. The purified CS was finally frozen at −80 °C, freeze-dried for 24 h (LIO-5P 4K, 5Pascal, Milan, Italy), and stored in a desiccator until use.

### 2.3. Formulation of RBCS Nanocomposites

Self-assembled RBCS nanocomposites were prepared via electrostatic interaction between RB and CS. Hereafter, this formulation is referred to as RBCS. A 3 mL RB solution (1.5 or 0.75 mg/mL, pH ≈ 6) was added dropwise to 3 mL of a CS solution (15 or 20 mg/mL in 1% *v*/*v* acetic acid, pH ≈ 3.5), according to the ratios reported in Table 1. The addition was performed gradually over 30 min under continuous stirring (700 rpm, room temperature), using a syringe (0.5 × 16 mm needle) positioned approximately 5 cm above the CS solution surface. Following this process, RBCS were obtained and further characterized.

RBCS were analyzed by photon correlation spectroscopy (PCS) (Coulter nanosizer N5, Beckman-Coulter Inc., Miami, FL, USA) to determine dimensional heterogeneity. RBCS were diluted with filtered MilliQ water (regenerated cellulose syringe filter, 0.20 μm pore size) to ensure light-scattering intensity within the required range. The analysis conditions were fluid refractive index 1.333; temperature 25 °C; viscosity 0.890 cP; angle of measurement 90°; sample time 3.0 ms; and sample run time 300 s.

### 2.4. Formulation of RBCS-Loaded AlgBs (RBCS-AlgBs)

Optimal unloaded AlgBs were first identified. AlgBs were prepared by dropping 4 mL of an aqueous alginate solution (20 mg/mL, pH ≈ 7) into a static 30 mL CaCl_2_ solution (pH ≈ 6) contained in a Petri dish. The alginate solution was dispensed using a syringe fitted with a 0.5 × 16 mm needle, held 10 cm above the surface of the CaCl_2_ solution. AlgBs formed immediately upon contact with CaCl_2_; afterwards, AlgBs were collected, frozen at −80 °C, and freeze-dried for 24 h. The influence of CaCl_2_ concentration on AlgB formation was evaluated using the parameters in Table 2.

**Figure 2:**
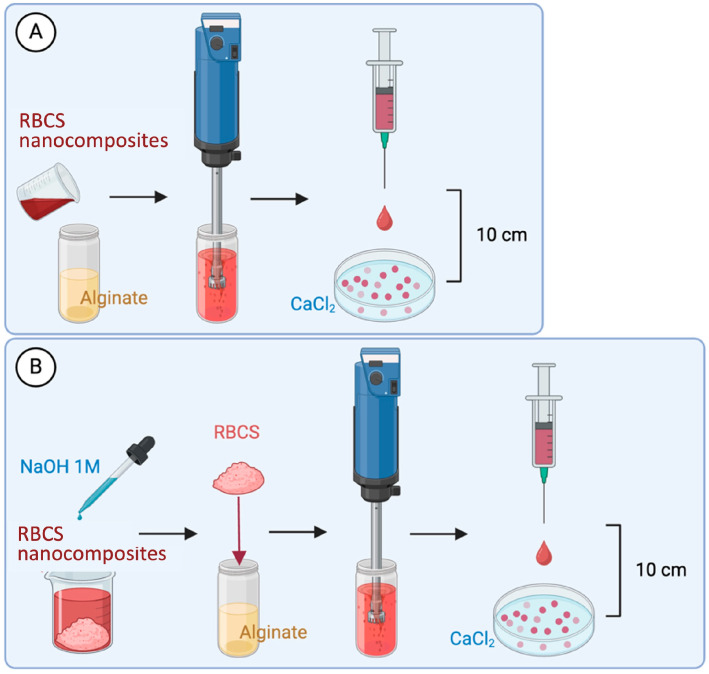
Methods tested for preparing RBCS-AlgBs. Method (**A**): RBCS were homogenized with alginate and dropped in CaCl_2_ solution. Method (**B**): RBCS were isolated by basification and centrifugation, homogenized with alginate solution, and dropped in CaCl_2_ solution. Created with Biorender.com.

Two methods (A and B) were tested to prepare RBCS-AlgBs (Figure 2). In method A, 4 mL of alginate solution (20 mg/mL, pH ≈ 7) were mixed with RBCS and homogenized using an Ultra-Turrax T25 Digital Homogenizer (IKA, Staufen, Germany) at 18,000 rpm for 1 min. The resulting dispersion was then dropped into 30 mL of a static CaCl_2_ solution (5 mg/mL, pH ≈ 6) contained in a Petri dish, using a syringe fitted with a 0.5 × 16 mm needle, held 10 cm above the surface of the CaCl_2_ solution. The resulting formulation was referred to as RBCS-AlgB(A). In method B, RBCS were first basified to pH 9 using 1 M NaOH to remove unreacted components, then centrifuged (10 min, 4000 rpm). The resulting pellet was isolated and re-dispersed in 4 mL of alginate solution (20 mg/mL, pH ≈ 7), followed by Ultra-Turrax homogenization (1 min, 18,000 rpm). The mixture was then dropped into static CaCl_2_ solution as described in Method A, yielding RBCS-AlgB(B). As controls, pure CS-loaded AlgBs (CS-AlgBs) were prepared using method B without the addition of RB, and pure RB-loaded AlgBs (RB-AlgBs) were prepared by dissolving RB (0.56 mg/mL) directly into 4 mL of alginate solution (20 mg/mL), followed by the same gelation procedure. All AlgBs were isolated, frozen at −80 °C and freeze-dried for 24 h prior to characterization.

### 2.5. Characterization of RB Delivery Systems

#### 2.5.1. Drug Content and Drug Loading

RBCS-20, RBCS20-AlgBs, and RB-AlgBs were analyzed for theoretical drug content (Theor. DC%), experimental DC% (Exp. DC%) and RB loading (DL%).

Theor. DC% was calculated as in Equation (1).

(1) $$Theor.\;DC\%=\frac{RB\;weighed}{Exp.\;weight\;of\;formulation}\times 100$$

Exp. DC% and DL% for RBCS-20 were determined following an indirect method. Briefly, RBCS-20 were basified to pH 9 (NaOH 1M) and centrifuged (10 min, 4000 rpm). The supernatant, containing unreacted RB, was analyzed by UV–Vis spectrophotometry (Shimadzu UV-1800, Kyoto, Japan) referencing a calibration curve in deionized water (pH 9) (calibration standards in the 0.625–10 µg/mL range; wavelength = 549 nm; y = 0.0692x + 0.0239; R^2^ = 0.9994). Exp. DC% and DL% were calculated as Equations (3) and (4), respectively.

(2) $$Exp.\;DC\%=\frac{RB\;\left(mg\right)\;weighed-RB\;\left(mg\right)\;in\;the\;supernatant}{Exp.\;weight\;\left(mg\right)\;of\;formulation}\times 100$$

(3) $$DL\%=\frac{RB\;\left(mg\right)\;weighed-RB\;\left(mg\right)\;in\;the\;supernatant}{RB\;\left(mg\right)\;weighed}\times 100$$

RB-AlgBs and RBCS20-AlgBs were analyzed using the same method. In these cases, the CaCl_2_ solution collected after bead isolation was analyzed to quantify the amount of RB not incorporated into the beads.

#### 2.5.2. Production Yield

AlgBs, RBCS-20, RBCS20-AlgBs, RB-AlgBs, and CS20-AlgBs were analyzed for the production yield (PY%) calculated according to Equation (4).

(4) $$PY\%=\frac{Exp.\;weight\;of\;RB\;delivery\;system}{Theor.\;weight\;of\;RB\;delivery\;system}\times 100$$

The Exp. weight of RB delivery systems was determined by weighing RBCS or beads after freeze-drying. The theor. weight of RB delivery systems was calculated as the sum of the all components used.

#### 2.5.3. Microscopy-Based Evaluations

RBCS-20, RBCS20-AlgBs, and RB-AlgBs were visually examined for morphology using optical microscopy (Leica EZ4W stereomicroscope, Leica Microsystems, Milton Keynes, UK) and scanning electron microscopy (SEM) (TM3030, Hitachi, Krefeld, Germany). With optical microscopy, samples before and after freeze-drying were examined. For SEM, only freeze-dried samples were analyzed, deposited onto adhesive carbon tape, and observed in low vacuum mode with a voltage of 15 kV without sample pre-treatment.

#### 2.5.4. Physicochemical Characterization

AlgBs, CS20-AlgBs, RBCS20-AlgBs, RBCS-20 and raw materials were analyzed for thermal properties using a Q100 differential scanning calorimeter (TA Instruments, Bellingham, WA, USA). Scans were performed from 25 °C to 300 °C with a heating rate of 10 °C/min under a constant flow of nitrogen (50 mL/min). A Q500 Thermogravimetric analyzer (TA Instruments, Bellingham, WA, USA) was used to further characterize the same materials. During the analyses, samples were heated from 10 °C to 500 °C at a rate of 10 °C/min under a nitrogen flow rate of 40 mL/min. Furthermore, the Fourier transform infrared spectra were recorded using a Spectrum Two FT-IR Spectrometer (Perkin Elmer, Waltham, MA, USA) equipped with a MIRacle™ diamond attenuated total reflectance (ATR) accessory (PIKE Technologies, Fitchburg, MA, USA). Each spectrum was recorded from 4000 cm^−1^ to 600 cm^−1^ with a resolution of 4 cm^−1^, and an average of 32 scans were collected.

### 2.6. In Vitro Drug Release Studies

Freeze-dried RBCS-20, RB-AlgBs and RBCS20-AlgBs were tested for RB release under a simulated GI environment. To do this, two conditions were experimented (Table 3). In the first one, buffer solutions at different pH values were used as acceptor mediums, and in the second experiment, simulated gastrointestinal fluids (SGFs) in fasted conditions were employed [23].

#### 2.6.1. Release Study in Buffer Solutions as Acceptor Mediums

The release study in buffer solutions was designed to test pH levels of 1.5, 4.5, and 7.2 (Italian Pharmacopoeia, XII edition). This study was conducted using a USP dissolution apparatus equipped with paddles (DT 70, Erweka, Langen, Germany) over a 24 h period. Conditions were set to a constant temperature of 37 °C and an agitation speed of 100 rpm. Sink conditions were assumed based on literature values for the aqueous solubility of the drug, which is reported to be up to 100 mg/mL [24]. In our system, the RB concentration was kept below 10% of this value to ensure sink conditions were maintained throughout the release experiments. First, the pH 1.5 solution was prepared to mimic the gastric environment and was composed of NaCl and 0.2 M HCl; 200 mL of this solution was placed into the dissolution basket and left to equilibrate to 37 °C before starting the test. Afterwards, the RB delivery systems were separately added to the dissolution basket (RB = 0.5 mg). After 1 h, the pH of the medium was adjusted to 4.5 by adding Trizma base (2.28 g/L) and anhydrous sodium acetate (1.77 g/L). This transition aimed to mimic passage into the upper small intestine. Following an additional 2 h, the pH was further adjusted to 7.2 by adding Trizma base (2.28 g/L) and anhydrous sodium acetate (1.77 g/L) to simulate lower intestinal and colonic conditions; the pH 7.2 remained constant until the end of the experiment. Throughout the 24 h, 1 mL samples were collected at predetermined intervals and immediately replaced by an equivalent volume of fresh buffer. The samples were analyzed by UV–Vis spectrophotometry following basification to pH 9 with NaOH (wavelength = 549 nm; y = 0.0692x + 0.0239).

#### 2.6.2. Release Study in SGFs as Acceptor Mediums

The release in SGFs was designed to simulate GI conditions in the fasted state to assess the initial release behavior of the formulations under standardized and reproducible conditions [23]. This study was conducted using an incubator with constant agitation at 100 rpm and temperature control at 37 °C. Sink conditions were maintained during this study. First, the simulated gastric medium (SGM, pH 1.6) was prepared containing 80 µL of STC, 20 µM lecithin, 0.1 mg/mL pepsin, and 34.2 mM sodium chloride; 10 mL of this solution was placed into a vial and left to equilibrate to 37 °C before starting the test. Afterwards, the RB delivery systems were separately added to the vials (RB = 0.1 mg). After 1 h, the medium was replaced with 10 mL of simulated intestinal medium (SIM, pH 6.5) containing 3 mM sodium taurocholate, 0.2 mM lecithin, 19.12 mM maleic acid, 34.8 mM sodium hydroxide, and 68.62 mM sodium chloride. The release study continued for an additional 23 h under the same conditions. Throughout the 24 h, 0.5 mL samples were collected at predetermined intervals and immediately replaced by an equivalent volume of fresh medium. The samples were analyzed by UV–Vis spectrophotometry following basification to pH 9 with NaOH (wavelength = 549 nm; y = 0.0692x + 0.0239).

#### 2.6.3. Data Analysis of In Vitro Release Profiles

The in vitro release profiles were analyzed by plotting the cumulative percentage of RB released (RB%) as a function of time for each RB delivery system. The cumulative amount of drug released at each time point was calculated using the Equation (5):

(5) $$Q_{t}=C_{t}\times V_{0}+\sum_{i=1}^{t-1}C_{i}\times V_{i}$$

where *Q_t_* is the cumulative amount of RB released at time *t*, *C_t_* is the RB concentration in the acceptor medium at each time point, *V*_0_ is the total volume of the acceptor medium, *C_i_* is the RB concentration in the sample at each previous time point, *V_i_* is the volume of the sample collected at each time point.

To assess the release mechanisms, the release data were fitted using linear regression to two standard kinetic models: zero-order and Korsmeyer–Peppas.

For the zero-order model, cumulative RB% released was plotted as a function of time and fitted to the Equation (6):

(6) $$\frac{M_{t}}{M_{\infty }}=K_{ZO}\times t$$

where *Mt*/*M*∞ is RB release fraction at time *t*, and *K_ZO_* is the zero-order release constant.

Lastly, in the case of the Korsmeyer–Peppas model, the cumulative RB% released was plotted against the square root of time and fitted to the Equation (8):

(7) $$\frac{M_{t}}{M_{\infty }}=K_{KP}\times t^{n}$$

where *Mt*/*M*∞ is RB release fraction at time *t*, *K_KP_* is the Korsmeyer–Peppas constant, and the value of the exponent *n* depends on the drug release mechanism [25,26].

### 2.7. Swelling Studies

The swelling behavior of freeze-dried RBCS20-AlgBs and RB-AlgBs was assessed under conditions simulating GI environments. Formulations were weighed, and size was measured with a digital microscope. Samples were then immersed in SGFs and periodically removed at designated intervals. Surface fluid was carefully removed with filter paper, and the swollen beads were immediately weighed and measured to monitor changes over time.

### 2.8. Statistical Analysis

All numerical results in this study are presented as mean ± standard deviation (SD) based on a minimum of n ≥ 3 experimental measurements unless otherwise specified. Data analysis, including statistical significance tests, was conducted using GraphPad Prism^®^ version 10.0.0 (153) (GraphPad^®^ Software, San Diego, CA, USA). The specific statistical methods used for individual data sets are detailed in Section 3. A *p*-value of less than 0.05 was considered indicative of statistical significance. The significance levels are visually represented: * *p* < 0.05, ** *p* < 0.01, *** *p* < 0.001, **** *p* < 0.0001.

## 3. Results and Discussion

This study aimed to develop and evaluate a delivery system for the oral administration of RB. Due to its hydrosoluble and ionizable nature, RB presents formulation challenges that limit its therapeutic potential. To overcome these limitations, a simple and well-established biopolymeric strategy was adopted: RBCS were prepared and subsequently incorporated into conventional AlgBs. While both components are widely used in drug delivery, their combination was investigated here as a practical approach for facilitating RB oral administration.

### 3.1. Formulation and Characterization of RBCS

RBCS were obtained via self-assembly through natural electrostatic interactions between positively charged CS and negatively charged RB, eliminating the need for additional chemicals and potentially enhancing the formulation’s safety profile. Various drug delivery systems have widely exploited CS’s ability to form polyelectrolyte complexes with oppositely charged compounds [27,28]. Such modifications alter the solubility and reactivity of native drugs, allowing them to respond to pH variations in the GI tract [29].

**Figure 3:**
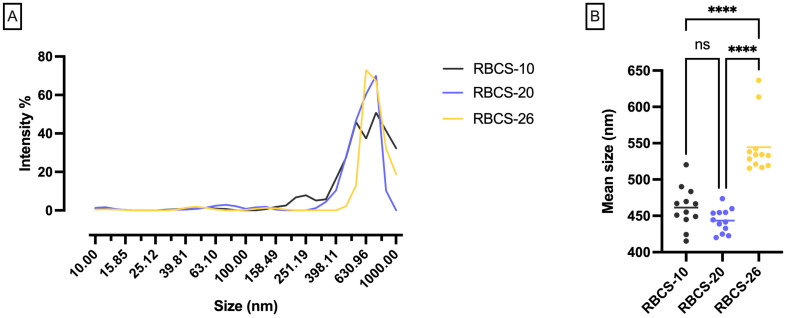
RBCS particle size distribution. (**A**) Size distribution data. (**B**) Scatter plot showing the mean particle size (nm) for each RBCS, with statistical analysis performed using one-way ANOVA and Tukey’s post hoc test (**** *p*-value < 0.0001) (n = 12).

To ensure high purity and remove protein contaminants, CS was purified before RBCS formulation. Various RB-to-CS ratios were tested, and the resulting RBCS were analyzed for their dimensional profiles (Figure 3). Among the tested RBCS, RBCS-20 exhibited the most uniform size distribution, with a mean particle size of 443 ± 16 nm and a coefficient of variation (CV%) of 4%, indicating high uniformity. In comparison, RBCS-10 had a mean size of 461 ± 28 nm (CV% = 6%), while RBCS-26 showed a larger mean size of 544 ± 39 nm (CV% = 7%). Based on its low CV%, RBCS-20 were selected as the lead RBCS.

No statistically significant differences were observed between RBCS-10 and RBCS-20; however, RBCS-26 differed significantly from both (*p*-value < 0.0001), indicating that the RB-to-CS ratio influences RBCS formation. Specifically, as the RB-to-CS ratio increased, particle size and sample heterogeneity tended to increase. A possible explanation for this trend is the presence of unreacted CS in solution, as RB molecules may have fully reacted, leaving excess unbound CS [30,31].

The selected RBCS-20 were further characterized for PY%, Exp. DC% and DL% to evaluate formulation efficiency. The Exp. DC% (5.6 ± 0.6%) was slightly lower than the theoretical value (Theor. DC% = 6.6 ± 0.6%), suggesting minor drug loss during formulation. As a result, DL% was calculated as 87 ± 4%. Additionally, the PY% (78 ± 7%) demonstrated that the self-assembly process was efficient, with minimal material loss.

**Figure 4:**
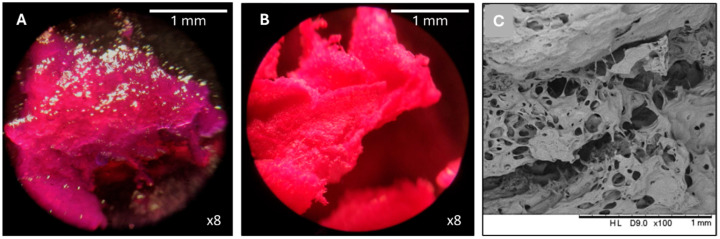
Microscopy characterization of RBCS-20. (**A**) non-freeze-dried RBCS-20, (**B**) freeze-dried RBCS-20 and (**C**) freeze-dried RBCS-20 by SEM.

Optical microscopy and SEM were used to compare the morphology of freeze-dried and non-freeze-dried samples (Figure 4). Non-freeze-dried samples exhibited a dense and amorphous structure, whereas freeze-dried samples displayed a highly porous network with surface irregularities, indicating structural collapse due to water loss. Although such changes are typical of freeze-dried polymeric systems and do not compromise the integrity of the dry formulation, complete rehydration upon administration is essential to restore the functional properties of RBCS-20, such as controlled drug release under GI conditions.

**Figure 5:**
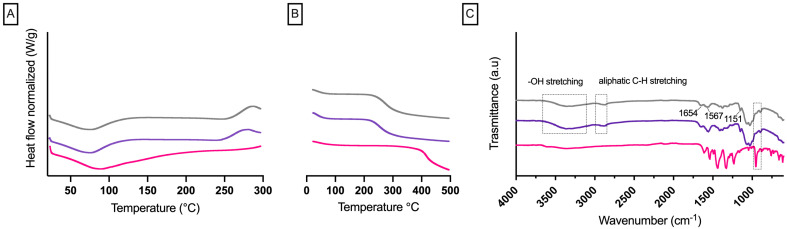
Physicochemical characterization of RBCS-20 and raw materials. (**A**) DSC thermograms. (**B**) TGA thermograms. (**C**) FT-IR spectra.

The physicochemical properties of RBCS-20 were evaluated through DSC, TGA and FTIR analysis to confirm molecular interactions between RB and CS (Figure 5).

DSC analysis (Figure 5A) showed a broad endothermic peak at ~84 °C for RB, attributed to water loss [32], and a similar peak for CS at ~88 °C, consistent with its hygroscopic nature [33]. These observations were supported by TGA analysis (Figure 5B), which revealed a corresponding mass loss of ~23% for RB and 26% for CS, confirming moisture evaporation. For RBCS-20, the RB endothermic peak became broader and less intense, while TGA showed a slightly lower overall mass loss (~20%) compared to CS, suggesting that RB-CS interactions modified the water retention properties of the system.

FTIR analysis (Figure 5C) further confirmed the formation of RBCS-20. The characteristic RB peaks at 1618 cm^−1^ (C=O stretching), 1331 cm^−1^ (C=C stretching), and 955 cm^−1^ (C–I bonding) remained visible in RBCS-20 but exhibited notable shifts. Specifically, the 955 cm^−1^ band of RB shifted to 896 cm^−1^, suggesting hydrogen bonding interactions between RB’s functional groups and CS’s amino groups. As described in our previous work [32], RB demonstrated the ability to interact through hydrogen bonds with the polymer polyvinyl alcohol. Although the excipients in this study are different, the type of shifts observed in FT-IR suggests a hydrogen bond interaction between RB and CS. Similarly, CS exhibited characteristic absorption bands at 1567 cm^−1^ and 1654 cm^−1^, corresponding to amide (CONH_2_) groups, which were preserved but slightly altered in RBCS-20, indicating intermolecular interactions. The lack of additional peaks suggests that RB is integrated into the CS matrix via electrostatic interactions and hydrogen bonding.

#### 3.1.1. In Vitro Release Profiles of RBCS-20

**Figure 6:**
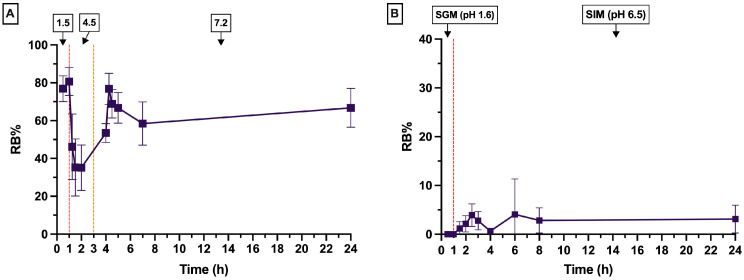
In vitro release profiles of RBCS-20. (**A**) Release in buffer solutions. (**B**) Release in SGFs.

The in vitro drug release behavior of RBCS-20 was evaluated under two different conditions: (1) buffer solutions with stepwise pH adjustments and (2) SGFs in the fasted state, containing bile salts and digestive enzymes (Figure 6).

In buffer solutions, RBCS-20 exhibited a burst release, with 77 ± 7% of RB released within 30 min, followed by a stabilization at 67 ± 10% over 24 h (Figure 6A). This biphasic release pattern suggests an initial phase of surface desorption of loosely bound RB molecules, followed by a slower, diffusion-driven release from the CS matrix. Conversely, in SGFs, RBCS-20 showed minimal release (3 ± 3% RB over 24 h) and poor reproducibility (CV% = 92%) (Figure 6B). This low and erratic drug release suggests that the presence of bile salts and digestive enzymes significantly alters the release behavior. The comparison between buffer and SGF conditions highlights CS’s sensitivity to bile salts, which can disrupt the release dynamics. At pH 6.5, near CS pKa (~6.3–6.5), partial protonation of amino groups (–NH_3_^+^) allows for strong electrostatic interactions with anionic bile salts (e.g., STC), which possess negatively charged sulfonate groups. These interactions can lead to CS chain condensation and reduced polymer flexibility, thereby increasing matrix density and limiting RB diffusion [34]. Additionally, bile salts may interact not only electrostatically with CS but also hydrophobically with RB itself, potentially altering its solubility and retention within the RBCS matrix. In parallel, bile salts can self-assemble into micelles capable of solubilizing and entrapping RB, thus reducing its free concentration and diffusional availability [35]. Zwitterionic surfactants, such as lecithin, may further stabilize these micelles, while partially ionized components like maleic acid can modulate ionic strength, indirectly influencing the release environment [36]. While these mechanisms remain hypothetical, they are consistent with literature reports on bile salt-polymer and bile salt-drug interactions.

Mathematical modeling was applied to analyze the release kinetics; however, the low R^2^ values indicated a poor fit, suggesting that RBCS-20 release is not well-described by conventional kinetic equations. This poor fit was also confirmed statistically, as none of the models produced significant non-zero slopes (*p*-value > 0.05), consistent with the highly variable release behavior observed for RBCS-20.

In conclusion, RBCS-20 represent a self-assembled nanocomposite system for RB delivery, achieving high drug loading and efficient production yield. Physicochemical analyses confirmed strong RB-CS interactions, supporting the formation of a stable complex. However, in vitro release in SGFs was minimal and poorly reproducible, highlighting the impact of bile salts and digestive enzymes on CS behavior and RB diffusion. To address these limitations and improve release performance under GI conditions, RBCS-20 were further encapsulated into AlgBs.

### 3.2. Formulation and Characterization of RBCS-AlgBs

To identify the optimal CaCl_2_-to-alginate ratio for AlgB formation, unloaded AlgBs were prepared and evaluated in terms of PY%. AlgB formation relies on electrostatic interactions between divalent calcium ions (Ca^2^^+^) and negatively charged carboxylate groups of alginate, which induce rapid gelation and matrix cross-linking. As shown in Table 4, AlgB-1.8 yielded 44 ± 3% with a low variability of 6.4%, indicating efficient and reproducible AlgB formation. In contrast, AlgB-3.75 and AlgB-5.6 showed significantly lower PY% (31 ± 3% and 17 ± 2%, respectively) and higher variability (CV% = 9.1% and 10.6%). These results suggest that higher Ca^2^^+^ may disrupt uniform cross-linking, leading to premature or uneven gelation and poor bead integrity. Based on its superior efficiency and reproducibility, the 1.8:1 CaCl_2_-to-alginate ratio was selected for the subsequent formulation of RBCS20-AlgBs.

To optimize the incorporation of RBCS-20 into AlgBs, two methods were tested. In Method A, RBCS-20 were directly mixed with the alginate solution and homogenized prior to gelation. However, this approach failed to produce structurally stable beads. The likely cause was electrostatic interference among the multiple charged species present, namely negatively charged alginate, unreacted CS, unbound RB, and dispersed RBCS-20, which disrupted the Ca^2+^ mediated gelation process [37,38]. This is consistent with previous findings where the simultaneous presence of multiple polyelectrolytes hindered the formation of uniform cross-linked matrices [39]. In contrast, Method B included a purification step in which RBCS-20 were basified to pH 9, centrifuged, and isolated from unreacted RB and CS. The purified RBCS-20 were then redispersed in alginate and subjected to the same gelation procedure. By removing unreacted components and reducing the overall charge competition of the system, Method B enabled more effective electrostatic interactions between alginate and Ca^2^^+^, resulting in uniform and structurally stable RBCS20-AlgBs [38]. Given these findings, Method B was selected as the lead formulation process for RBCS20-AlgBs.

RBCS20-AlgBs, RB-AlgBs, and their corresponding unloaded AlgBs were characterized for PY%, Exp. DC% and DL% to assess formulation efficiency (Table 5).

RBCS20-AlgBs exhibited a significantly higher DL% (96 ± 1%) compared to RB-AlgBs (47 ± 12%), reflecting the enhanced encapsulation efficiency imparted by CS. The Exp. DC% was also nearly doubled (2.4 ± 0.3% vs. 1.3 ± 0.4%), suggesting that the pre-complexation of RB with CS promoted its retention within the AlgB matrix and improved compatibility with the polymer network [40,41]. This stabilizing effect is further supported by the significantly higher PY% of RBCS20-AlgBs (73 ± 11%) compared to RB-AlgBs (37 ± 2%). Conversely, the absence of CS in RB-AlgBs led to reduced DL%, greater variability, and lower reproducibility, most likely due to weaker electrostatic interactions [40,42]. Additionally, the competition between RB and alginate for Ca^2+^-binding sites, combined with the rapid onset of gelation, may have contributed to the lower encapsulation efficiency observed in RB-AlgBs [40,41]. Notably, no significant differences in PY% were observed between RB-loaded and unloaded AlgBs, indicating that drug incorporation did not negatively affect bead formation.

**Figure 7:**
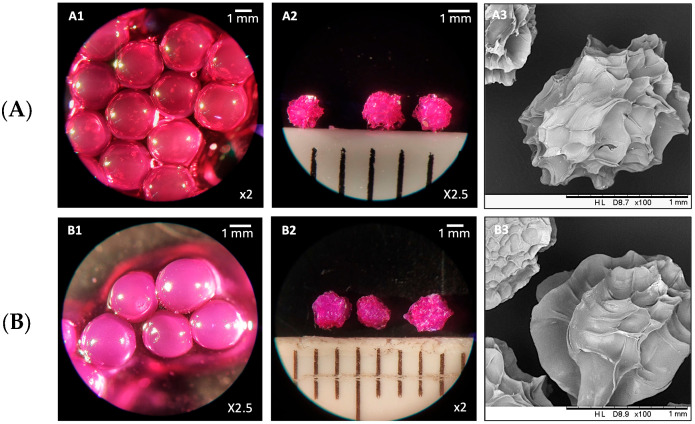
Microscopy characterization of different RB-loaded AlgBs. (**A**) RB-AlgBs: (**A1**) non-freeze-dried, (**A2**) freeze-dried and (**A3**) freeze-dried by SEM. (**B**) RBCS20AlgBs: (**B1**) non-freeze-dried, (**B2**) freeze-dried and (**B3**) freeze-dried by SEM.

Morphological characterization was performed using optical microscopy and SEM to evaluate the structural qualities of RB-AlgBs and RBCS20-AlgBs in both non-freeze-dried and freeze-dried states (Figure 7). In their non-freeze-dried form, both AlgBs exhibited a smooth and spherical morphology. After freeze-drying, SEM images revealed an apparently non-porous structure, with surface resembling rose petal-like formations. Notably, non-freeze-dried RBCS20-AlgBs appeared opalescent, an optical property consistent with the Tyndall effect, caused by light scattering in the colloidal CS network [43]. This phenomenon was not observed in RB-AlgBs, suggesting that the presence of CS modified the optical properties of the AlgBs.

**Figure 8:**
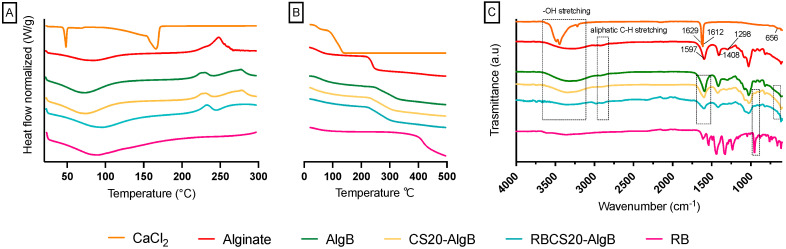
Physicochemical characterization of different RB-loaded AlgBs and raw materials. (**A**) DSC thermograms. (**B**) TGA thermograms. (**C**) FT-IR spectra.

The physicochemical properties of RBCS20-AlgBs were analyzed using DSC, TGA, and FTIR to assess the effects of RBCS-20 encapsulation within the AlgB matrix (Figure 8).

The DSC thermogram of alginate (Figure 8A) showed a broad endothermic peak at ~85 °C, attributed to water loss, and an exothermic decomposition peak at ~254 °C, which was confirmed by TGA analysis (Figure 8B), showing a 50% mass loss between 215 and 260 °C [44]. The CaCl_2_ DSC curve displayed a sharp melting peak at 49 °C, followed by an endothermic peak at 166 °C, associated with its decomposition [45]. TGA analysis indicated corresponding mass losses of ~10% and ~80%, confirming these thermal transitions.

For RBCS20-AlgBs, the disappearance of the CaCl_2_ melting peak in the DSC curve suggests interactions between CS, alginate, and Ca^2^^+^ during bead formation, leading to a modified thermal profile. Additionally, the thermal behavior of RBCS20-AlgBs remained largely similar to its individual components, displaying a broad endothermic peak near 90 °C, indicative of water loss, and decomposition occurring between 230 °C and 300 °C, as confirmed by TGA data showing ~80% mass loss.

FT-IR analysis (Figure 8C) provided further evidence of molecular interactions in RBCS20-AlgBs. The alginate spectrum exhibited a broad O–H stretching band (3000–3600 cm^−1^), aliphatic C–H stretching (2913 cm^−1^), and carboxylate (–COO^−^) asymmetric and symmetric stretching at 1597 cm^−1^ and 1408 cm^−1^, respectively. The CaCl_2_ spectrum showed characteristic O–H stretching (3600–3000 cm^−1^), H–O–H bending at 1629 cm^−1^ and 1612 cm^−1^, and a prominent Ca–O stretching peak at 656 cm^−1^ [46,47]. In RBCS20-AlgBs, the sharp alginate peak at 1597 cm^−1^ shifted to lower wavenumbers, likely due to the substitution of Na^+^ by Ca^2^^+^ during gelation, altering the polymer’s ionic environment. Additionally, RB exhibited a peak shift from 955 cm^−1^ to 938 cm^−1^ in RBCS20-AlgBs, indicating hydrogen bonding and structural rearrangement within the alginate-CS matrix. Furthermore, some of RB’s characteristic peaks were partially masked by the strong absorption bands of excipients, further supporting its successful encapsulation.

These findings confirm that RB successfully interacted with both CS and alginate during AlgB formation, with Ca^2^^+^-induced cross-linking playing a key role in stabilizing the polymer network. The integration of RBCS-20 into AlgBs preserved its physicochemical properties while enhancing the structural stability of the final system, making it a promising candidate for controlled oral drug delivery applications.

#### In Vitro Swelling and Release Profiles of RBCS20-AlgBs and RB-AlgBs

**Figure 9:**
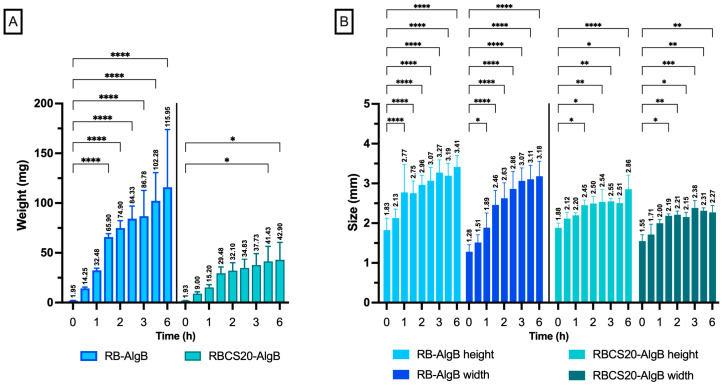
Swelling behavior of RB-AlgBs and RBCS20-AlgBs under simulated GI conditions over 6 h (n = 4). (**A**) Change in size and (**B**) weight over time. Data were analyzed using one-way ANOVA with Tukey’s post hoc test. Statistical significance * *p* < 0.05; ** *p* < 0.01 *** *p* < 0.001; **** *p* < 0.0001.

Swelling studies in simulated GI conditions assessed the water absorption capacity of RB-AlgBs and RBCS20-AlgBs (Figure 9).

**Figure 10:**
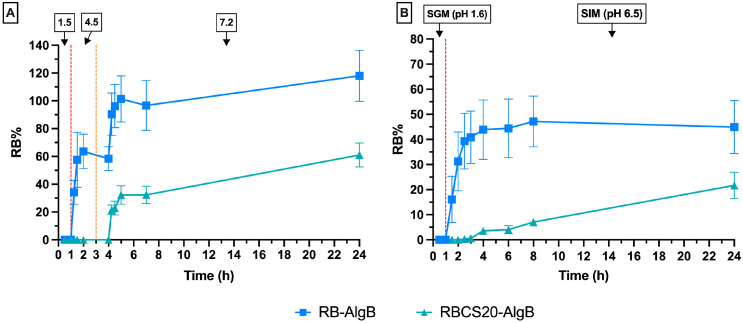
In vitro release profiles of RB-AlgBs and RBCS20-AlgBs. (**A**) Release in buffer solutions. (**B**) Release in SGFs.

Both AlgBs exhibited progressive increases in size and weight over 6 h. RB-AlgBs showed substantial swelling, with its weight rising from 1.95 ± 0.20 mg to 87 ± 26 mg, and dimensions increasing from ~2 mm to ~3 mm (height) and ~1 mm to ~3 mm (width) within the first 3 h. However, RB-AlgBs lost structural integrity and disintegrated by 6 h in the intestinal phase (SIM), indicating insufficient mechanical stability. In contrast, RBCS20-AlgBs exhibited a more controlled and gradual swelling profile. Weight increased only from 1.92 ± 0.20 mg to 43 ± 18 mg and size ranging from ~2 mm to ~3 mm (height) and ~1.5 mm to ~2 mm (width) throughout this study. This behavior suggests that the incorporation of RBCS-20 contributed to maintaining the structural integrity of the alginate matrix. This effect can be attributed to electrostatic interactions between CS’s cationic amino groups (–NH_2_) and the anionic groups of RB. These interactions reduce the availability of free hydrophilic sites (–NH_2_) for water uptake, limiting excessive swelling. Additionally, the presence of CS contributed to matrix reinforcement, counteracting the disintegration observed in RB-AlgBs [48]. Overall, the limited and controlled swelling of RBCS20-AlgBs may be considered advantageous, as it reduces the risk of burst and favors sustained drug release. These results, together with the release profiles discussed in Figure 10, support the functional benefits of integrating RBCS into AlgBs to achieve more stable and predictable performance under GI conditions.

In buffer solutions, RB-AlgBs exhibited a rapid burst release, with 90 ± 15% of RB released within the first 4 h, indicating that alginate alone is insufficient to sustain drug release (Figure 10A). As expected, no drug release occurred during the initial hour at pH 1.5 due to the protonation of alginate, which leads to the formation of a compact alginic acid gel that restricts swelling and drug diffusion. Upon transition to higher pH, alginate deprotonation triggered matrix solubilization and disintegration, resulting in the observed burst release. In contrast, RBCS20-AlgBs demonstrated a more gradual and controlled release profile. Only 22 ± 4% RB was released at 4 h, increasing to 61 ± 9% at 24 h, underscoring the stabilizing effect of CS. Electrostatic interactions between CS and alginate limited swelling at acidic pH, while CS itself acted as a structural reinforcement throughout the release process. This dual effect delayed drug diffusion and minimized burst release, especially at elevated pH where alginate alone would dissolve rapidly [48].

A similar trend was observed in SGFs. RB-AlgBs released 45 ± 10% of RB over 24 h, with a major release event at 6 h (41 ± 26%), likely due to matrix swelling and disintegration in the intestinal phase (SIM, pH 6.5). After this peak, the release plateaued, showing no significant increase between 6 and 24 h (*p*-value > 0.05). Conversely, RBCS20-AlgBs maintained a linear and sustained release, reaching 22 ± 5% over 24 h, highlighting its enhanced resistance to GI degradation [49]. When comparing the two systems across both conditions, the most notable differences were observed for RBCS20-AlgBs, which was more sensitive to the complex SGF environment, likely due to CS-bile salt interactions, as discussed in Section 3.1.1. On the other hand, RB-AlgBs displayed similar release behavior in both media, suggesting that alginate, being fully anionic at pH 6.5, interacts weakly with bile salts and enzymes. These findings demonstrate that encapsulating RBCS-20 into AlgBs significantly improved the performance of the delivery system. Compared to RBCS-20 alone, RBCS20-AlgBs overcame the limitations of low and erratic release in SGFs and excessive burst in buffer conditions. Moreover, when compared to RB-AlgBs, RBCS20-AlgBs showed superior drug retention and structural stability, confirming the synergistic role of RB-CS pre-complexation within the alginate matrix.

Due to the nature of the release curves in SIM, mathematical modelling was applied to gain insight into the release mechanism (Table 6). The Korsmeyer–Peppas and zero-order models were employed. When these models were applied to the entire release profile of RB-AlgBs, neither provided a good fit. This is unsurprising, given the plateau observed in the release profile of this formulation. However, when the plateau section (release after 2 h) was excluded from the analysis, both models showed a good fit, with the Korsmeyer–Peppas model displaying slightly higher R^2^ values. The obtained n value of 0.77 suggests that the release mechanism is a combination of Fickian diffusion and matrix relaxation, which is consistent with typical behavior observed in hydrogel-based matrices [26]. In contrast, for RBCS20-AlgBs, the entire release profile was used for modelling. The best fit was observed with the zero-order model, which aligns with the n exponent obtained from the Korsmeyer–Peppas model, indicating that the primary mechanism of release is matrix relaxation.

Previous studies on other AlgB systems have reported predominantly diffusion-driven mechanisms, particularly in densely cross-linked matrices [50,51]. However, in this case, bile salt interactions are likely to hinder free drug diffusion. These results are consistent with previously reported findings on bile salt interactions. Such interactions are more pronounced in formulations containing CS, thereby limiting RB diffusion and resulting in zero-order drug release. In contrast, RB-AlgB formulations did not show the same tendency to form complexes with bile salts as previously reported, and therefore RB diffusion remains observable.

## 4. Conclusions

This study investigated a novel oral delivery system for RB, addressing its poor stability and bioavailability in the GI tract. The two-step formulation strategy, first self-assembling RBCS-20 nanocomposites, then embedding them into AlgBs, proved essential to achieving effective oral release. While RBCS-20 alone exhibited high DL% and structural stability, it failed to ensure reproducible and sustained release under simulated GI conditions. Instead, RB encapsulation into AlgBs alone (RB-AlgBs) resulted in premature drug release and structural disintegration. Only the integration of preformed RBCS-20 into AlgBs successfully overcame both limitations, producing a system with gastroresistance, mechanical robustness, and controlled intestinal release. These results highlight the conceptual advancement of this work: the development of a hybrid system in which RB-CS complexation enhances drug stability and entrapment, while the AlgB matrix modulates release kinetics. The synergistic effect of both components was essential for achieving sustained and reproducible delivery, supporting this strategy as a promising platform for oral RB administration. Future studies will include cell-based and in vivo evaluations to validate the system’s safety and therapeutic potential. These investigations will be critical to confirming its clinical relevance and supporting translational advancement.

## Figures and Tables

**Table 1:** Formulation parameters tested during the formulation of RBCS.

Sample	RB Solution (mg/mL)	CS Solution (mg/mL)	CS/RB Ratio
RBCS-10	1.5	15	10:1
RBCS-20	0.75	15	20:1
RBCS-26	0.75	20	26.6:1

**Table 2:** Formulation parameters tested during the formulation of AlgBs.

Sample	CaCl_2_ Solution (mg/mL)	CaCl_2_/Alginate Ratio
AlgB-1.8	5	1.8:1
AlgB-3.75	10	3.75:1
AlgB-5.6	15	5.6:1

**Table 3:** Experimental conditions for the in vitro drug release studies.

Parameter	Condition 1 Buffer Solutions ^1^	Condition 2 Simulated Gastrointestinal Fluids ^2^
Amount of RB	0.5 mg	0.1 mg
Volume of Release Medium	200 mL	10 mL
Temperature	37 °C	37 °C
Agitation Speed	100 rpm	100 rpm
Apparatus	USP dissolution apparatus (paddles)	Incubator
pH Conditions	1.5 (1 h), 4.5 (2 h), 7.2 (21 h)	1.6 (1 h), 6.5 (23 h)
Gastric Medium Composition	NaCl, HCl	STC ^3^, Lecithin, Pepsin, NaCl, pH 1.6
Intestinal Medium Composition	NaCl, HCl, Trizma base, sodium acetate	STC, Lecithin, Maleic acid, NaOH, NaCl, pH 6.5
Sample Volume Collected	1 mL	0.5 mL

^1^ Italian Pharmacopoeia, XII edition. ^2^ Fasted states. ^3^ STC = sodium taurocholate.

**Table 4:** Production yield of AlgBs (n = 3).

Sample	PY% (Mean ± SD)	CV%
AlgB-1.8	44 ± 3 ° *	6.42
AlgB-3.75	31 ± 3 ° #	9.11
AlgB-5.6	17 ± 2 * #	10.56

Data were analyzed with ordinary one-way ANOVA, and Tukey’s test for multiple comparisons: ° and # *p*-value < 0.01; * *p*-value < 0.0001. PY% = production yield. CV%= variation coefficient.

**Table 5:** Production yield, drug content and drug loading of different RB-loaded and corresponding unloaded AlgBs (n ≥ 3).

Sample	PY% (Mean ± SD)	Theor. DC% (Mean ± SD)	Exp. DC% (Mean ± SD)	DL% (Mean ± SD)
AlgB	44.0 ± 3.0	-	-	-
RB-AlgB	37.0 ± 2.0 *	2.6 ± 0.2 °	1.3 ± 0.4 °	47.0 ± 12.0 #
CS20-AlgB	68.0 ± 4.0	-	-	-
RBCS20-AlgB	73.0 ± 11.0 *	2.5 ± 0.3	2.4 ± 0.3	96.0 ± 1.0 #

Data were analyzed with ordinary one-way ANOVA, and Tukey’s test for multiple comparisons: *, °, # *p*-value < 0.0001. PY% = production yield Theor. DC% = theoretical drug content Exp. DC% = experimental drug content. DL% = drug loading.

**Table 6:** Results obtained after fitting RB release in SIM from AR-AlgBs and RBCS20-AlgBs to different release models.

	Zero Order		Korsmeyer–Peppas		
	R^2^	K_ZO_ (h^−1^)	R^2^	K_KP_ (h^−n^)	n
RB-AlgBs (all curve)	0.213	0.010	0.842	0.327	0.149
RB-AlgBs (linear section)	0.980	0.266	0.994	0.294	0.769
RBCS20-AlgBs (all curve)	0.991	0.010	0.988	0.008	1.06

## Data Availability

Data are contained within the article.
